# Efficacy and Safety of Vildagliptin and Remogliflozin as Add-on Therapy to Metformin in Patients of Type 2 Diabetes Mellitus: An open-label comparative study

**DOI:** 10.18295/squmj.1.2024.003

**Published:** 2024-05-27

**Authors:** Vikram Sharma, Shalini Chawla, Sandeep Garg, Bhupinder Singh

**Affiliations:** 1 Department of Pharmacology, Maulana Azad Medical College & Associated Hospitals, New Delhi, India; 2 Department of Internal Medicine, Maulana Azad Medical College & Associated Hospitals, New Delhi, India

**Keywords:** Remogliflozin, Vildagliptin, Metformin, Type 2 Diabetes Mellitus, Efficacy, Safety, Glycaemic Control, Weight Loss, India

## Abstract

**Objectives:**

This study aimed to evaluate the safety and efficacy of remogliflozin compared to vildagliptin as an add-on drug to metformin in type 2 diabetes mellitus (T2DM) treatment. Metformin is considered a first-line drug in T2DM. However, as the disease progresses with heightened insulin resistance and declining β-cell function, the use of metformin alone is often inadequate to achieve optimum glucose levels.

**Methods:**

This prospective, randomised study was conducted at Maulana Azad Medical College and Associated Hospital in New Delhi, India, between February 2020 to January 2021. This study recruited 60 T2DM patients aged 35–70 years with glycated haemoglobin (HbA1c) >6.5% taking metformin at a daily dosage of 1,500–3,000 mg for ≥3 months. Patients were randomly assigned in a 1:1 ratio to receive either vildagliptin (50 mg) or remogliflozin (100 mg) twice daily for 90 days. The primary endpoint was a change in HbA1c levels from baseline to the end of 90 days whereas secondary endpoints were changes in lipid profile and weight.

**Results:**

The decrement in mean HbA1c levels was significantly higher in the remogliflozin group than in the vildagliptin group (−8.1% versus −2.4%; *P* <0.001). In addition, more significant weight loss was found in remogliflozin-treated patients (−5.2% versus −0.6%; *P* <0.01). Both treatments were well tolerated throughout the study.

**Conclusion:**

Compared to vildagliptin, remoglilflozin was significantly more effective in glycaemic control and weight loss in patients with T2DM and can therefore be considered as an add-on drug in T2DM not adequately controlled by metformin monotherapy.

**Advances in Knowledge**

- *The decrement in mean glycated haemoglobin (HbA1c) levels was significantly higher in the remogliflozin group than in the vildagliptin group. Moreover, remogliflozin was superior to vildagliptin in reducing mean body weight with both treatments being well-tolerated.*
- *This study is distinctive where the efficacy and safety of remogliflozin, a novel sodium-glucose cotransporter subtype-2 (SGLT2) inhibitor is compared with vildagliptin, a commonly prescribed dipeptidyl peptidase-4 (DPP4)-inhibitor as an add-on therapy to metformin in patients with type 2 diabetes mellitus (T2DM).*

**Application to Patient Care**

- *Remoglilflozin is significantly more effective than vildagliptin in glycaemic control and has more significant weight loss potential as an add-on drug to metformin in the treatment of patients with T2DM. Thus, remoglilflozin can potentially be used as an add-on drug in obese patients with T2DM not adequately controlled by metformin monotherapy.*

Type 2 diabetes mellitus (t2dm) is a prevalent chronic condition worldwide, leading to a rise in both illness and death rates.^1^ In 2010, approximately 6.4% of adults, totalling 285 million individuals, were affected by diabetes, and this figure is predicted to grow to 7.7% encompassing 439 million people globally by 2030.^1^ Notably, India recorded an estimated 62.4 million diabetic patients in 2011, with projections indicating a staggering increase to 101.2 million cases by 2030.^1^ The current guidelines for the comprehensive management of T2DM advocate a patient-focused strategy to determine the appropriate pharmacological treatments.^2^ Apart from achieving optimal glycaemic control, several other factors affect the selection of anti-diabetic agents, including their impact on body weight, the risk of causing hypoglycaemia and the presence of other comorbidities.^2^ T2DM is a gradually advancing condition that necessitates the intensification of treatment over time to maintain glycaemic control.^3^ Metformin is considered a first-line drug for the management of T2DM.^3^ Nevertheless, as the disease progresses, characterised by increased insulin resistance and decreased β-cell function, relying solely on metformin often proves insufficient in attaining the optimum glucose level.^3^ Since metformin acts by enhancing insulin sensitivity, the addition of therapy utilising an insulin-independent pathway may be beneficial.^3^

The joint position statement of the American Diabetes Association and European Association for the Study of Diabetes recommends the usage of 1 of the 6 commonly employed antihyperglycaemic agents containing sulfonylurea, thiazolidinedione, dipeptidyl peptidase-4 (DPP4) inhibitor, sodiumglucose cotransporter subtype-2 (SGLT2) inhibitor, glucagon-like peptide-1 (GLP-1) receptor agonist or basal insulin analogue as an add-on therapy when the glycated haemoglobin (HbA1c) target of ≤6.5% is not attained following 3 months of treatment with metformin alone.^4^ DPP4 inhibitors and SGLT2 inhibitors are widely used therapies for T2DM that are associated with a low incidence of hypoglycaemia.^5^ DPP4 inhibitors are body-weight neutral, whereas SGLT2 inhibitors promote weight loss and reduce systolic blood pressure.^6^^,^^7^ According to the 2017 American Association of Clinical Endocrinologists and American College of Endocrinology comprehensive glycaemic control algorithm, SGLT2 inhibitors are higher than DPP4 inhibitors in the recommended order of use, both as standalone therapy and as an addon treatment in the management of T2DM.^8^

Vildagliptin, a potent and selective inhibitor of DPP4, improves glycaemic control by increasing the availability of endogenous incretin hormones, GLP-1 and glucose-dependent insulinotropic polypeptide.^9^ Complementing the pharmacological effect of metformin, vildagliptin enhances glucose-dependent insulin secretion and suppresses glucagon release, thereby improving glycaemic control and contributing to weight-neutrality and reduced hypoglycaemia.^10^ Remogliflozin, a novel SGLT2 inhibitor, is to be administered as prodrug remogliflozin etabonate.^11^ Inhibition of SGLT2 (which is selectively expressed in the proximal convoluted tubules of the kidney) leads to increased excretion of glucose in the urine, resulting in reduced blood glucose concentrations and has therapeutic benefits in T2DM.^12^ The recommended dose of remogliflozin etabonate for the treatment of T2DM in India is 100 mg twice daily.^13^

The current study hypothesised that remogliflozin may be non-inferior to vildagliptin in the treatment of type 2 diabetes mellitus as an add-on therapy to metformin. In light of this, this study aimed to determine whether there was any change in HbA1c from baseline to the end of 90 days and secondarily whether any changes were seen in the lipid parameters and body weight, relative to baseline.

## Methods

This prospective, randomised, open-label, parallel-group, interventional and comparative study was conducted at the medicine outpatient department (OPD) at Maulana Azad Medical College and Associated Hospital in New Delhi, India. Patient enrolment into the study began in February 2020 and ended in January 2021.

The inclusion criteria were as follows: (1) patients with a diagnosis of T2DM with HbA1c >6.5% (48 mmol/mol); (2) those taking metformin at a dosage of 1,500–3,000 mg/day for ≥3 months; (3) those aged between 35–70 years of both genders; and (4) those who provided written informed consent to participate in the study. The following exclusion criteria were used: (1) patients with type 1 diabetes or secondary diabetes; (2) those taking any other glucose-lowering agents other than metformin; (3) those with hepatic dysfunction (AST or ALT ≥2.5 times upper normal limit [UNL] or bilirubin >2 times of UNL); (4) those with renal dysfunction (estimated glomerular filtration rate as per Modification of Diet in Renal Disease formula <45 mL/min/1.73 m^2^); (5) those with genitourinary tract infections; (6) those with lower limb cellulitis or ulcer; (7) patients with a known case of osteoporosis; (8) patients allergic to the study medications; (9) those who were pregnant or breastfeeding; and (10) those who did not give consent.

Eligible patients were divided randomly into two groups in a 1:1 ratio. One group received vildagliptin (50 mg; twice daily), while the other group received remogliflozin (100 mg; twice daily), both as additional medication to their existing metformin intake at a dosage of between 1,500–3,000 mg/day, for 90 days. The randomisation process utilised a computer-based dynamic allocation method to ensure a balanced distribution of key baseline characteristics, such as age, gender, metformin dose, HbA1c levels, lipid profile and body weight.

During patient recruitment, a comprehensive medical history was gathered and a thorough general and systemic examination was conducted with a particular focus on identifying any potential complications related to T2DM. Additionally, the patients underwent various essential investigations, including liver and kidney function tests, routine urine examination, HbA1c, lipid profile, fundus examination and electrocardiogram. After the initial assessment, the patients were scheduled for a follow-up visit after 90 days. During the follow-up visit, they underwent similar examinations and investigations as performed at recruitment. All the relevant details were carefully recorded in a pre-designed clinical proforma for accurate documentation and analysis.

Throughout the study, patient well-being was closely monitored for any adverse events (AEs) via telephonic communication and regular in-person visits to the medicine OPD where they received their prescribed treatment drugs. The patients were assured that they could reach out to the researchers at any time if they experienced any form of discomfort during the study. Any AEs that occurred were documented. Additionally, the researchers maintained regular contact with the patients, checking on their well-being and ensuring they adhered to the prescribed treatment and instructions.

The collected data were transformed into variables, coded and entered into Microsoft Excel, 2019 (Microsoft, Redmond, Washington, USA). The data were analysed and statistically evaluated using Statistical Package for Social Sciences (SPSS), Version 25 (IBM Corp., Armonk, New York, USA). The quantitative data were expressed in mean ± standard deviation, and differences between the two groups were tested by student’s t-test (unpaired) or Mann Whitney U test for normal and non-normal data, respectively. The qualitative data were expressed in frequency and percentage; differences between the proportions were tested by Fisher’s exact test or Chi-squared test for parametric and non-parametric distributions, respectively. A *P* value of <0.05 was considered statistically significant. The safety analysis included all treated patients.

This study was registered with the Clinical Trials Registry of India (CTRI/2020/02/023120). The study protocol was approved by the institutional ethics committee of Maulana Azad Medical College, New Delhi in November 2019 (F.1/IEC/MAMC/(70/05/2019/No 559). Before the initiation of the study, written informed consent was obtained from all the patients involved in the study. Privacy was maintained during data collection and subjects were ensured of complete confidentiality about the information they share in the study.

## Results

**Figure 1:**
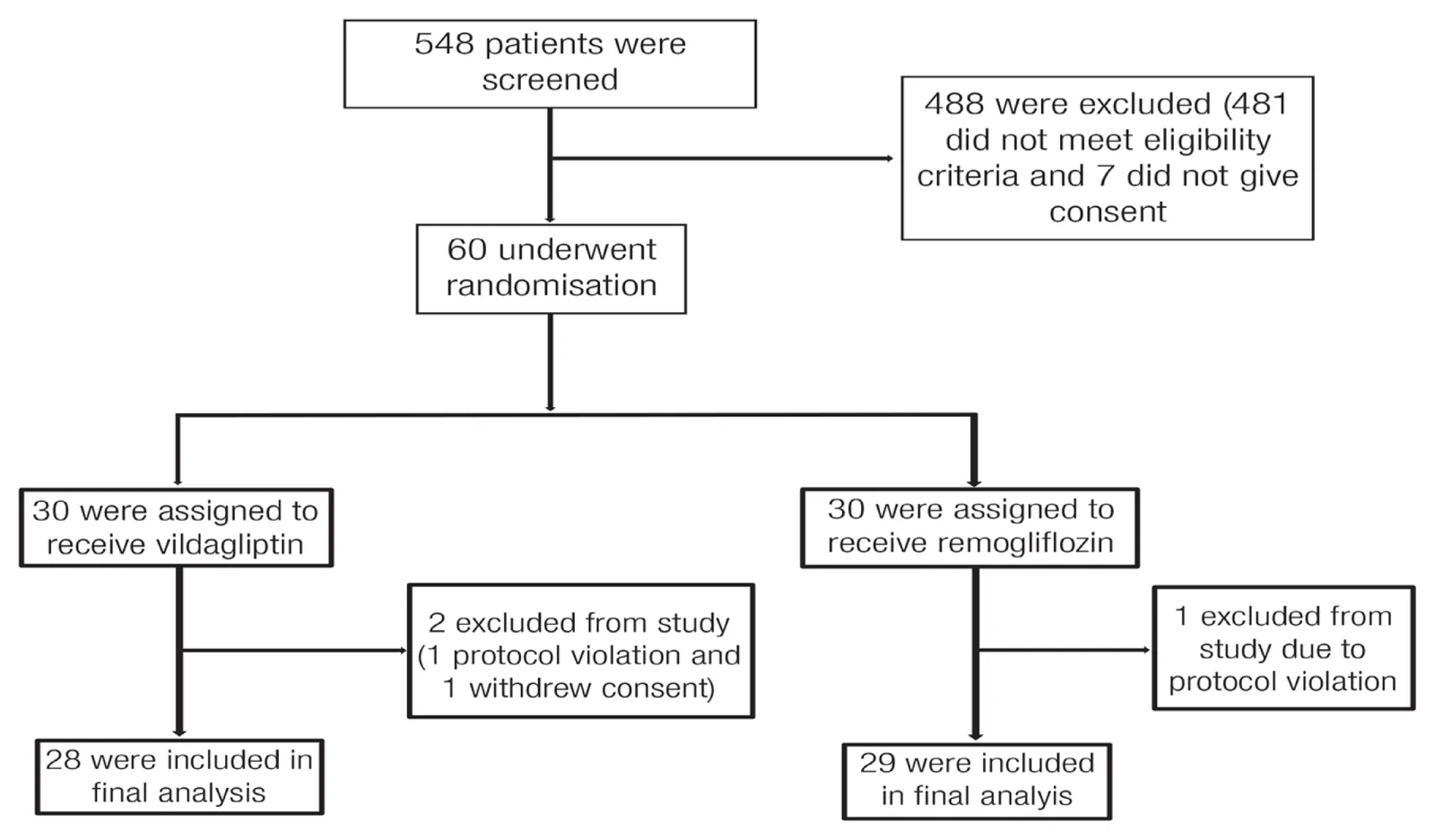
Flowchart showing patient enrolment, allocation and analysis.

A total of 548 patients were screened, out of which 488 were excluded (481 did not meet eligibility criteria and 7 did not give consent). Finally, 60 patients were enrolled and randomised, and 57 completed the study and were included in the final analysis. Among them, 28 and 29 patients were in the vildagliptin and remogliflozin groups, respectively [Figure 1]; 2 patients (1 in each group) were excluded after randomisation due to a protocol violation as they started taking glucose-lowering agents other than the study medications. The baseline demographic, clinical and laboratory characteristics of the study population were comparable between both treatment groups [Table 1].

**Figure 2:**
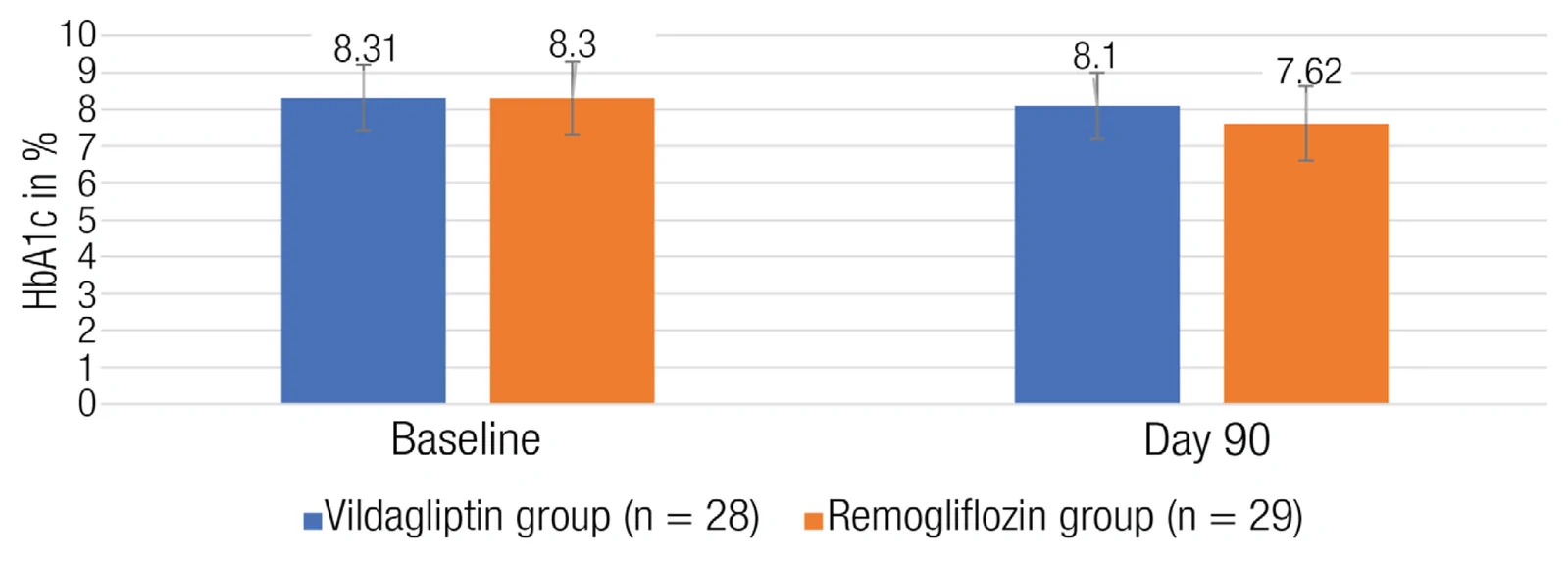
Comparison of glycated haemoglobin level at baseline and day 90 between the treatment groups. *HbA1c = glycated haemoglobin*.

The improvement in the HbA1c levels was significantly more pronounced in the remogliflozin group than in the vildagliptin group after 90 days of treatment (−0.67 ± 0.24 versus −0.20 ± 0.22%; *P* <0.001) [Table 2 and Figure 2]. The weight loss was also significantly more in the remogliflozin group than in the vildagliptin group relative to the baseline levels (−3.73 ± 1.91 versus −0.4 ± 1.52 kg; *P* <0.01) [Table 3]. Regarding the lipid parameters, there was a significant decrement in total cholesterol (−2.33 ± 9.54 versus 6.47 ± 4.85 mg/dL; *P* = 0.001), triglycerides (−1.1 ± 9.32 versus 6.3 ± 6.1 mg/dL; *P* < 0.01), low-density lipoprotein (LDL; −1.70 ± 7.78 versus 4.13 ± 3.57 mg/dL; *P* = 0.02) and very low-density lipoprotein (VLDL; −0.27 ± 5.22 versus 4.07 ± 3.6 mg/dL; *P* <0.01) levels in the remogliflozin group compared to the levels in the vildagliptin group. The increment in high-density lipoprotein (HDL) level in the remogliflozin group was also significantly more pronounced compared to the vildagliptin group (1.30 ± 4.63 versus −1.6 ± 3.27 mg/dL; *P* = 0.03) [Table 3].

**Figure 3:**
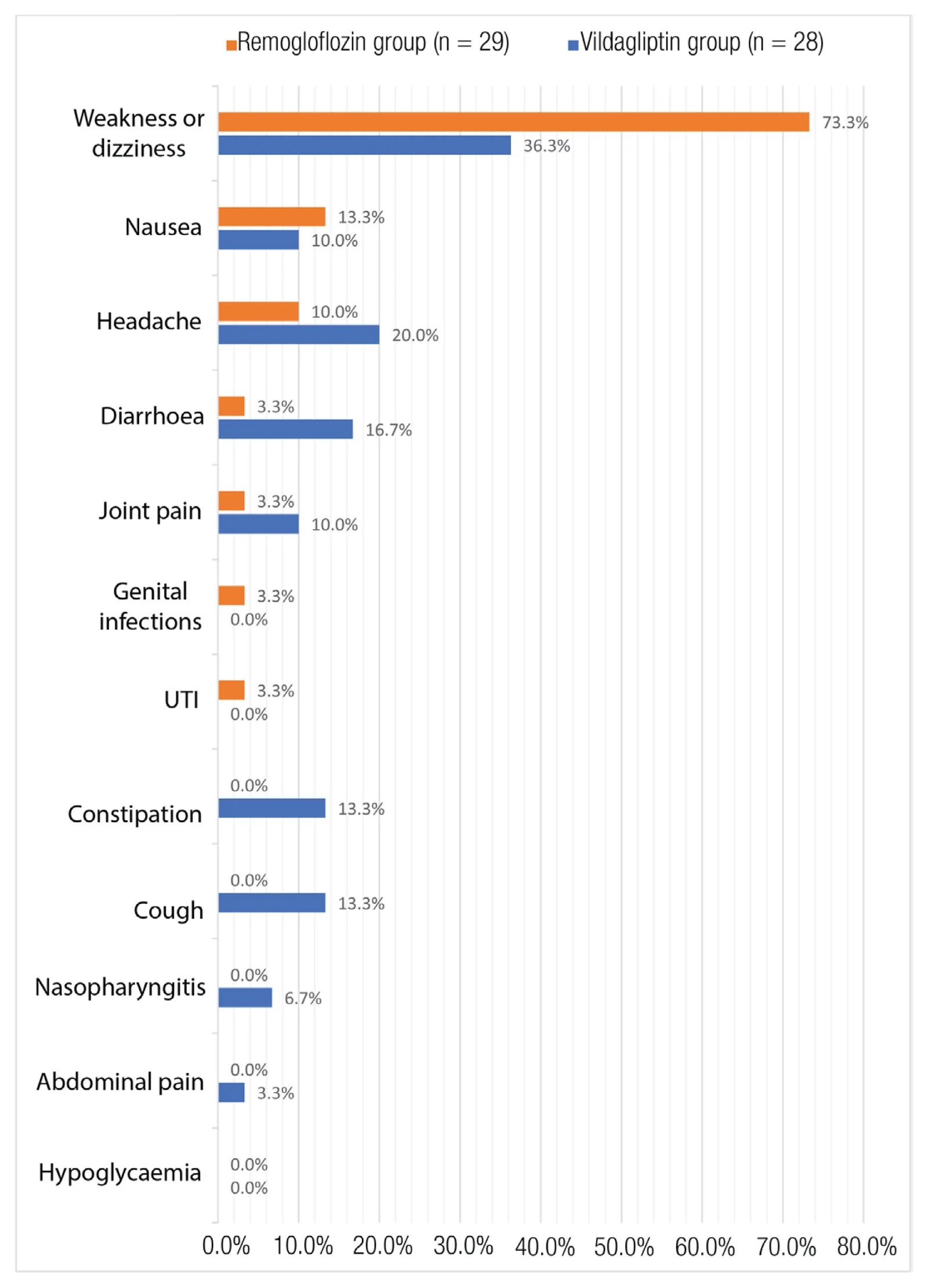
Comparison of adverse events between the treatment groups. *UTI = urinary tract infection*.

During the study, 19 of 28 patients (67.9%) in the vildagliptin group and 17 of 29 patients (58.6%) in the remogliflozin group reported AEs. The nature of AEs was mild such as dizziness or weakness, nausea, headache, diarrhea, joint pain, genital infection, urinary tract infection, constipation, cough, nasopharyngitis and abdominal pain [Figure 3]. Most of the AEs were self-limiting and resolved spontaneously during the study period. Thus, the treatment protocol was not altered. No subjects in either group were withdrawn because of AEs. No significant differences in AEs were found between the groups. No serious AEs including hypoglycaemia were observed in either group.

## Discussion

This prospective randomised study aimed to evaluate the efficacy and safety of remogliflozin, a novel SGLT2 inhibitor in comparison to vildagliptin, a commonly prescribed DPP4 inhibitor in the treatment of T2DM. The study enrolled 60 patients with T2DM with inadequate glycaemic control (average HbA1c level: 8.30% or 67 mmol/mol) on metformin alone. After 90 days of treatment, this study found that remogliflozin, compared to vildagliptin as an add-on therapy to metformin, was superior to vildagliptin in terms of glycaemic control, lipid-lowering potential and weight loss capacity. Both medications were well tolerated and no serious AEs were observed during the study.

A randomised, double-blind, active- and placebo-controlled trial was conducted to evaluate the efficacy and safety of twice-daily remogliflozin etabonate for the treatment of T2DM.^14^ In that 90-day study, 336 treatment-naive subjects with T2DM and an HbA1c between 7.0 to 9.5% were randomised to remogliflozin etabonate (50 mg, 100 mg, 250 mg, 500 mg or 1000 mg twice daily), matching placebo or 30 mg pioglitazone once daily.^14^ The results indicated that the twice-daily administration of remogliflozin etabonate led to a dose-dependent improvement in glycaemic control, with statistically significant reductions in body weight compared to the placebo group.^14^ Additionally, the treatment was generally well-tolerated by the participants.^14^ The current study also found that the group that administered remogliflozin 100 mg twice daily, after 90 days showed improvement in glycaemic control in terms of decrement in mean HbA1C levels from baseline by 8.1% comparable to the previous study, which showed a decrement by 11.9%.^14^ In terms of the effect on lipid profile, the study showed a 1.3% decrement in mean total cholesterol levels, a 1.5% decrement in mean LDL levels and a 0.9% decrement in mean VLDL levels.

The study findings were in contrast to the findings of a previous study by Sykes *et al*., which showed increments in total cholesterol, LDL and VLDL levels by 2.5%, 4.9% and 1.2% respectively.^14^ This can be attributed to the limitations of this study, which are small sample size and short duration. However, the study also found a 1.7% decrement in mean triglycerides levels and a 3% increment in mean HDL levels. These findings were comparable to a previous study by Sykes *et al*., which showed a 3.5% decrement in triglycerides level and a 6.5% increment in HDL levels.^14^ The overall changes in lipid profiles in the remogliflozin treatment group may in part reflect improvements in glycaemia and a change in insulin sensitivity, as insulin activates lipoprotein lipase to hydrolyse triglycerides, resulting in a decrease in triglycerides level, increase in HDL-cholesterol concentration and a shift in the processing of particles towards cholesterol-rich lower-density particles.^15^

A similar pattern of lipid changes has been documented with canagliflozin, reflecting a 2.0–6.1% increase in LDL cholesterol, a 6.1–6.8%, increment in HDL cholesterol and a decrement of 5.4–10.2% in triglycerides.^16^ Although the lipid-lowering potential of remogliflozin was statistically more compared to vildagliptin, the meagre increment makes it unsuitable to be used as an alternative to standard lipid-lowering drugs for treating T2DM patients with dyslipidaemia.

At the end of 90 days, patients in the current study receiving remogliflozin showed a statistically significant reduction in mean body weight from baseline at 5.2% comparable to a previous study, which showed a 5% reduction in body weight.^14^ Similar findings were also previously observed in the DIVERSITY-CVR trial where body weight loss of ≥3.0% was significantly achieved in the dapagliflozin group compared to sitagliptin.^17^ Reported AEs were mild and self-limiting in both the groups and comparable to findings in a previous study by Sykes *et al*. where the overall rate of AEs in the remogliflozin treatment groups did not differ from that in the placebo group and none were reported as serious.^14^ The current study did not observe any episode of hypoglycaemia in either group similar to findings in a previous study by Sykes *et al*. where no subjects in the remogliflozin or pioglitazone treatment groups were withdrawn because of hypoglycaemia or other AEs.^14^ These data indicate that both remogliflozin and vildagliptin can be used to improve glycaemic control while minimising hypoglycaemic episodes in the management of patients with T2DM.

The present study has a few limitations which should be noted. First, this was an open-label study and second, all patients were of Indian ethnicity as they were recruited from medicine OPD where patients were receiving drugs as part of their standard care. The Trial Evaluating Cardiovascular Outcomes with Sitagliptin reported that East Asians had the greatest HbA1c level response to sitagliptin, a DPP4 inhibitor of the same class as vildagliptin.^18^ Finally, the sample size of this study was small with a short duration of follow-up as it was planned as a pilot study. The sample size was not calculated and participant recruitment was done on that basis. Nonetheless, the promising results of this study have encouraged the researchers to further evaluate the potential of remogliflozin including its impact on renal and cardiovascular outcomes in T2DM. To further validate the generalisability of these findings, it is requisite to conduct future trials with a larger number of participants, adequate representation of different ethnicities and long-term observation.

## Conclusion

This is the first study to directly evaluate the efficacy and safety of remogliflozin compared to vildagliptin as an add-on therapy to metformin in patients with inadequately controlled T2DM. The study showed that remogliflozin was superior to vildagliptin in terms of glycaemic control after 90 days of treatment. Additionally, loss in body weight occurred more significantly in the remogliflozin group.

## Figures and Tables

**Table 1:** Characteristics of included type 2 diabetes mellitus patients at baseline to be treated with vildagliptin and remogliflozin (N = 57)

Characteristic	Mean ± SD or n (%)		*P* value*
	Vildagliptin group (n = 28)	Remogliflozin group (n = 29)	
**Age in years**	50.57 ± 10.01	49.10 ± 9.36	0.50
**Gender**			0.89
Males	14 (50.0)	15 (51.7)	
Females	14 (50.0)	14 (48.3)	
**Frequency of usage of metformin daily dosage in g**			0.86
1.5	6 (21.4)	5 (17.2)	
2	20 (71.4)	21 (72.4)	
2.5	2 (7.2)	3 (10.3)	
**HbA1c in %**	8.31 ± 0.92	8.30 ± 1.05	0.99
**Lipid parameters in mg/dL**			
Total cholesterol	198.67 ± 40.26	192.40 ± 36.85	0.28
Triglyceride	174.35 ± 55.68	166.37 ± 53.39	0.29
LDL	111.43 ± 21.12	115.33 ± 26.14	0.27
HDL	40.53 ± 8.22	43.10 ± 7.15	0.22
VLDL	34.27 ± 12.05	30.37 ± 12.57	0.11
**Body weight in kg**	65.27 ± 10.49	71.40 ± 14.03	0.09

SD = standard deviation; HbA1c = glycated haemoglobin, LDL = lowdensity lipoprotein; HDL = high-density lipoprotein; VLDL = very lowdensity lipoprotein.

* Using student t-test (unpaired) and Fisher’s exact test for continuous and categorical variables, respectively

**Table 2:** Comparison of mean change in glycated haemoglobin level from baseline to day 90 in the vildagliptin- and remogliflozin-treated groups (N = 57)

	HbA1c in % ± SD		*P value
	Vildagliptin group (n = 28)	Remogliflozin group (n = 29)	
Baseline	8.31 ± 0.92	8.30 ± 1.05	0.99
Day 90	8.10 ± 0.84	7.62 ± 1.00	0.05
Mean change	−0.20 ± 0.22	−0.67 ± 0.24	<0.001

HbA1c = glycated haemoglobin; SD = standard deviation.

* Using student t-test (unpaired)

**Table 3:** Comparison of mean change in parameters from baseline to day 90 in two treatment groups

Parameter	Mean parameter change in mg/dL ± SD		*P value
	Vildagliptin group (n = 28)	Remogliflozin group (n = 29)	
ΔTotal cholesterol	6.47 ± 4.85	−2.33 ± 9.54	0.001
ΔTG level	6.3 ± 6.1	−1.1 ± 9.32	<0.01
ΔLDL level	4.13 ± 3.57	−1.70 ± 7.78	0.02
ΔHDL level	−1.6 ± 3.27	1.30 ± 4.63	0.03
ΔVLDL level	4.07 ± 3.6	−0.27 ± 5.22	<0.01
ΔBody weight in kg	−0.4 ± 1.52	−3.73 ± 1.91	<0.01

SD = standard deviation; Δ = amount of change from baseline to day 90; TG = triglycerides; LDL = low-density lipoprotein; HDL = high-density lipoprotein; VLDL = very low-density lipoprotein.

* Using student t-test (unpaired)
